# Analysis of plant cell death-inducing proteins of the necrotrophic fungal pathogens *Botrytis squamosa* and *Botrytis elliptica*

**DOI:** 10.3389/fpls.2022.993325

**Published:** 2022-10-11

**Authors:** Michele C. Malvestiti, Maikel B. F. Steentjes, Henriek G. Beenen, Sjef Boeren, Jan A. L. van Kan, Xiaoqian Shi-Kunne

**Affiliations:** ^1^ Wageningen University, Laboratory of Phytopathology, Wageningen, Netherlands; ^2^ Wageningen University, Laboratory of Biochemistry, Wageningen, Netherlands

**Keywords:** *Botrytis* spp., plant-necrotroph interaction, host specificity, cell death-inducing proteins, synteny

## Abstract

Fungal plant pathogens secrete proteins that manipulate the host in order to facilitate colonization. Necrotrophs have evolved specialized proteins that actively induce plant cell death by co-opting the programmed cell death machinery of the host. Besides the broad host range pathogen *Botrytis cinerea*, most other species within the genus *Botrytis* are restricted to a single host species or a group of closely related hosts. Here, we focused on *Botrytis squamosa* and *B. elliptica*, host specific pathogens of onion (*Allium cepa*) and lily (*Lilium* spp.), respectively. Despite their occurrence on different hosts, the two fungal species are each other’s closest relatives. Therefore, we hypothesize that they share a considerable number of proteins to induce cell death on their respective hosts. In this study, we first confirmed the host-specificity of *B. squamosa* and *B. elliptica*. Then we sequenced and assembled high quality genomes. The alignment of these two genomes revealed a high level of synteny with few balanced structural chromosomal arrangements. To assess the cell death-inducing capacity of their secreted proteins, we produced culture filtrates of *B. squamosa* and *B. elliptica* that induced cell death responses upon infiltration in host leaves. Protein composition of the culture filtrate was analysed by mass spectrometry, and we identified orthologous proteins that were present in both samples. Subsequently, the expression of the corresponding genes during host infection was compared. RNAseq analysis showed that the majority of the orthogroups of the two sister species display similar expression patterns during infection of their respective host. The analysis of cell death-inducing proteins of *B. squamosa* and *B. elliptica* provides insights in the mechanisms used by these two *Botrytis* species to infect their respective hosts.

## Introduction

In the battle for survival, plant pathogenic fungi have evolved different strategies to colonize their hosts. While biotrophic fungi obtain nutrients from living host cells *via* specialized feeding structures, necrotrophs kill the cells of their host to acquire nutrients. For a long time, fungi with a necrotrophic lifestyle have been considered to invade their hosts in a rather unsophisticated manner. Using lytic and degradative enzymes, the fungus first kills and subsequently grows into host cells and eventually colonizes host tissue. In the past decades however, evidence has been accumulating that the interaction between necrotrophs and their hosts is more subtle and sophisticated than previously appreciated. Necrotrophs actively induce plant cell death by co-opting the programmed cell death machinery of the host using specialized effector proteins, to a certain extent similar to biotrophs (Dickman et al., 2001; van Kan, 2006). The induction of plant cell death using proteinaceous effectors is vital for the success of infection (Tan et al., 2010; Stergiopoulos et al., 2013; Veloso and van Kan, 2018). For some necrotrophic effectors the corresponding plant receptor proteins required for effector recognition and triggering of the cell death response have been identified (Schürch et al., 2004; Abeysekara et al., 2009; Gao et al., 2015; Shi et al., 2016). The cell death response to effectors of necrotrophs is based on similar mechanisms as the recognition of effectors from biotrophic pathogens, but leads to disease susceptibility instead of disease resistance as is the case for biotrophs (Lorang et al., 2007; Tan et al., 2010).

The role of effectors of necrotrophs in plant-pathogen interactions is best studied in Septoria nodorum blotch in wheat, caused by the Ascomycete *Parastagonospora nodorum*. The first identified effector from *P. nodorum* was SnTox1, a small secreted protein that induced necrosis upon infiltration in wheat lines carrying the sensitivity gene *Snn1* (Liu et al., 2004). SnTox1 knockout mutants lost virulence on *Snn1*-carrying wheat lines, and introduction of SnTox1 into an avirulent *P. nodorum* isolate conferred pathogenicity on wheat lines harbouring *Snn1* (Liu et al., 2012). By genetic analysis, more *P. nodorum* effectors and corresponding wheat sensitivity genes were identified such as SnToxA-*Tsn1*, SnTox4-*Snn4*, and SnTox6-*Snn6* (Abeysekara et al., 2009; Faris et al., 2010; Tan et al., 2012; Gao et al., 2015). To date, nine necrotrophic effectors and corresponding host sensitivity loci have been identified in the *P. nodorum*-wheat pathosystem (Duba et al., 2018; Cowger et al., 2020). The severity of infection depends in a quantitative manner on the number and identity of pathogen effectors and corresponding wheat sensitivity genes (Oliver et al., 2012; Phan et al., 2016; Ruud et al., 2017; Haugrud et al., 2019).

The broad host range model necrotroph *Botrytis cinerea* is also known to secrete proteins that induce cell death upon recognition by the host. *B. cinerea* endopolygalacturonases (PGs) induce cell death upon infiltration in *Arabidopsis thaliana*, but only in lines harbouring leucine-rich repeat (LRR) receptor-like protein RBPG1 (Zhang et al., 2014). Other proteinaceous virulence factors that function as cell death-inducing effectors in *B. cinerea* are BcXyl11a, BcXyl1 and BcXYG1, hemicellulases that induce necrosis in plant tissue independent from their catalytic activity (Noda et al., 2010; Zhu et al., 2017; Yang et al., 2018). Xylanases are recognized by the LRR receptor-like proteins *LeEix1* and *LeEix2* and the cell death response to BcXyl1 and BcXYG1 requires the LRR receptor-like kinases BAK1 and SOBIR1 (Ron and Avni, 2004; Zhu et al., 2017; Yang et al., 2018). In addition to the cell wall degrading enzymes with cell death-inducing activity, often considered as PAMPs or catalytic necrosis-inducing proteins (NIPs), *B. cinerea* also secretes cell death-inducing proteins that lack a known enzymatic domain and are referred to as effectors. Amongst others BcNep1, BcNep2, BcSpl1 and BcIEB1 have been heterologously produced and induced cell death upon infiltration in plant leaf tissue (Arenas et al., 2010; Frías et al., 2011; Frías et al., 2016).

The genus *Botrytis* comprises ~40 described species of which a few, including *B. cinerea*, are generalist plant pathogens with very broad host ranges (Hyde et al., 2014; Garfinkel et al., 2017). Most *Botrytis* species, however, are considered host-specific since they are pathogenic on a single host or a few taxonomically related hosts. An inventory by Mercier et al., 2019 of polyphagy indices of *Botrytis* species indicated that *B. elliptica* (commonly considered to be a pathogen specifically to lily and known as “lily fire blight”) was reported to infect as many as 21 host species from 10 distinct genera, and has a polyphagy index of 7.1. By contrast, its close relatives *B. deweyae, B. sinoalli* and *B. squamosa*, that cluster with *B. elliptica* in a subclade of the phylogenetic tree of the genus *Botrytis* (Valero-Jimenéz et al., 2020), have polyphagy indices below 1.5 (Mercier et al., 2019). In this study we focussed on *B. elliptica* and *B. squamosa*, two closely related sister species that are reported to cause disease on lily and onion, respectively. For both species, induction of programmed cell death is a key step in the infection process and it is achieved by means of secreted proteinaceous effectors (Van Baarlen et al., 2004; Malvestiti et al., 2021; Steentjes et al., 2022). We aimed to analyse whether the shared secreted proteins between these two closely related species can contribute to cell death induction in their respective host and non-host plant. We verified the host specificity and sequenced and assembled high quality genomes of *B. squamosa* and *B. elliptica* and analysed their synteny. We compared the cell death inducing capacity of the culture filtrates of *B. squamosa* and *B. elliptica*, and analysed the protein composition of these culture filtrates and the expression of the corresponding genes during host infection.

## Materials and methods

### Plant material and growth conditions

Bulbs of *Lilium* spp. cultivar “Asiatic” were planted in plastic crates containing potting soil and grown in a greenhouse under natural day light at a minimum night temperature between 12 and 15°C and maximum day temperature between 24 and 26°C. Mature leaves as described by Bar and Ori (2014) were harvested before the flowering stage and used for disease and infiltration assays.

*Nicotiana benthamiana* plants were grown in potting soil in a greenhouse with 16 h light at 24°C and 8 hours darkness at 22°C and 75% relative humidity. Both disease and infiltration assays were carried out on leaves that were still attached to the plant. *Allium cepa* (cv Ceresco F1) plants were grown from seeds in potting soil in a climate chamber with 12h light, 70% relative humidity, 18°C day temperature and 16°C night temperature. Fully grown leaves of 8 to 10 weeks-old onion plants were used for inoculation and infiltration assays. Disease assays were conducted on cut onion leaves, while for infiltration assays the leaves remained attached to the plant.

### Fungal material and growth conditions

*Botrytis squamosa* isolate MUCL31421 (Steentjes et al., 2021), *Botrytis elliptica* isolate 9401 (Malvestiti et al., 2021) and *Botrytis cinerea* isolate B05.10 (Van Kan et al., 2017) used in this research were stored as conidia suspensions in 20% glycerol at -80°C. To obtain conidia for inoculation, *B. elliptica* and *B. cinerea* were grown on Malt Extract Agar (50 g/L, Oxoid), whereas *B. squamosa* was grown on autoclaved onion leaves on top of water agar (Oxoid). Sporulation was induced by illumination with UV-A lamps. After harvesting, the conidia were collected and washed in demineralized water, counted with the Bürker-Türk counting chamber, adjusted to a concentration of 10^6^ conidia/mL and stored until use in darkness at 4°C.

### Disease assays

Freshly harvested conidia of *B. squamosa*, *B. elliptica* and *B. cinerea* were inoculated in 12 g/L Potato Dextrose Broth (Difco) in 2 μL droplets at a spore concentration of 10^5^ conidia/mL. Detached lily and onion leaves were placed on petri dishes in wet plastic boxes. Three lily leaves were abaxially inoculated with each fungus with four droplets per leaf avoiding leaf veins and leaf edges. Three detached onion leaves were first gently wiped with clean tissue paper to remove the epicuticular layer and then inoculated each with four droplets per fungus. Two *N. benthamiana* plants were kept in the pot and placed in boxes containing water. Three leaves per plant were adaxially inoculated with each fungus by applying four droplets on distinct sectors of the leaves, avoiding leaf veins and edges. Each plant-fungus combination was inoculated in a separate box. All boxes were closed with lids to maintain humid conditions and sealed with paper tape. The boxes were kept at room temperature under ambient light. After four days of incubation inoculated leaves were photographed and lesions were measured with a caliper. Lesion diameters were plotted using ggplot2 in R (Version 4.0.2).

### DNA extraction, genome assembly and annotation

High molecular weight genomic DNA for Nanopore sequencing was extracted from *in vitro* grown mycelium as described in Valero-Jiménez et al. (2020). Both genomes of *B. elliptica* and *B. squamosa* were assembled with Oxford Nanopore reads. The adapters of the reads were firstly trimmed with porechop, version 0.2.4 (https://github.com/rrwick/Porechop). NECAT (Chen et al., 2021) was then launched for genome assembly, with genome sizes of 48 Mb and 55 Mb for *B. elliptica* and *B. squamosa* respectively and other parameters were set as default. After the assembly, we polished the *B. elliptica* genome with Illumina short reads using Pilon (Walker et al., 2014) and the *B. squamosa* genome by aligning with the previously published genome assembly (Valero-Jiménez et al., 2020). Subsequently, the two genomes were aligned with MUMer 3.07 (Kurtz et al., 2004) using PROmer (-l 150, -c 500, -g 100, -b 200). The genome of *B. elliptica* was annotated using Funannotate (https://funannotate.readthedocs.io). The previously predicted secretome of *B. elliptic*a strain 9612 was subsequently mapped to the new genome assembly and the missing secretome genes in the new assembly were manually added. The previously published annotation of *B. squamosa* of the same strain was mapped to the new assembly.

### Production of culture filtrate, ammonium sulfate precipitation and leaf infiltration

*B. squamosa* and *B. elliptica* Culture Filtrate (CF) samples were obtained by growing each fungus in a 250 mL flask containing 50 mL of liquid medium with 3 g/L Gamborg B5 salts (Duchefa, Haarlem, Netherlands), 10 mM potassium phosphate pH=6.0, 0.1% D-Glucose and 5mL of lily or onion leaf extract in demi water, respectively. The leaf extract was obtained by grinding 30g of fresh harvested leaf material in 250 mL demineralized water. The homogenate was centrifuged (3500 rpm, 20 min) and the supernatant was concentrated by freeze drying. The concentrate was redissolved in 10mL water, filter-sterilized and added to the liquid medium. The liquid culture was inoculated with a spore concentration of 10^5^ conidia/mL medium. The cultures were incubated at 140 rpm, at 20°C in the dark. After 5 days of growth, the CF was passed through a layer of Miracloth (Calbiochem, San Diego, CA, United States), filter-sterilized (0.45 μm pore size, Millipore, Amsterdam, Netherlands) and kept on ice. A mock liquid medium was prepared without fungal conidia. Of the ca 40 mL harvested CF samples, 2mL were stored at 0°C for one night. To the remaining volume ammonium sulfate (LabChem, Tiel, Netherlands) was gradually added and mixed on a rolling bench at 4°C until the CFs were saturated with salt. Samples were centrifuged 30min at 4°C (4000rpm). After centrifugation a solid pellet of precipitated compounds was collected. The pellet was redissolved in 10mM potassium phosphate pH=6.0 and transferred to a prewetted dialysis membrane (Spectra/Por 3 Dialysis Membrane 3.5 kD MWCO, Repligen), and dialyzed overnight at 4°C in 5 L 10mM potassium phosphate pH=6.0. The dialyzed samples were collected and stored at 0°C until infiltration. The same plant genotypes used in the disease assays were tested for the infiltration assays. For lily, ca 100 μL of each sample (crude CFs, dialyzed pellets and mock liquid media) was infiltrated with a 1 mL syringe in two spots of two different detached leaves on their abaxial side. The leaves were placed in moist plastic boxes and incubated at 20°C under ambient light. The same volume of sample was infiltrated in two spots on two onion and *N. benthamiana* leaves whereby onion and *N. benthamiana* leaves were not detached from the plant. After 3 days incubation all leaves were photographed and the response was evaluated using red light imaging (Villanueva et al., 2021).

### Proteomic analysis

*B. elliptica* and *B. squamosa* proteins that were analyzed with mass spectrometry were sampled from the liquid culture as described in the above section. Proteins were prepared for mass spectrometry as described in **Supplementary Information 1**. The samples were subjected to LC–MS/MS according to the parameters described in (Xiong et al., 2020). For protein identification and quantification, each run with all MS/MS spectra obtained was analyzed with Maxquant 2.0.3.0 with the Andromeda search engine. The *B. squamosa* and *B. elliptica* annotated genomes were used for protein mapping. A maximum of two missed cleavages and a mass deviation of 20 ppm for the fragment MS/MS peaks were allowed. The false discovery rate (FDR) was set to 1% on both peptide and protein level. The length of peptides was set to at least seven amino acids. Protein identification required minimally two distinct peptides of which at least one unique and at least one unmodified. Intensity based absolute quantification (iBAQ) algorithm was calculated as the sum of all peptide intensities divided by the number of theoretically observable tryptic peptides.

### RNA extraction and gene expression analysis

For *B. elliptica*, RNAs used to create RNAseq library and to analyze gene expression profiles were isolated from 5 days old mycelial tissue of *B. elliptica* 9401 grown *in vitro* on MAE and from lily leaf tissue of cultivar “Asiatic” inoculated with conidia suspension of *B. elliptica* 9401 harvested at 16, 24 and 40 hours post inoculation (hpi), with three replicate samples per timepoint. RNA extraction was carried out from 100 mg freeze-dried, grinded material using the RNA extraction protocol of Maxwell^®^ RSC Plant RNA Kit (Product AS 1500, Promega). For *B. squamosa* RNAs were extracted from 5 days old mycelial tissue grown on MAE supplemented with onion extract and from onion leaf tissue inoculated with *B. squamosa* isolate MUCL31421 at 16, 24 and 48 hpi with three replicate samples per timepoint. 100 mg of freeze-dried grinded material was incubated with Trizol (Ambion, Life Technology) and treated with chloroform. Technical EtOH (Sigma Aldrich) was added to the aqueous phase and the mixture was used as input for RNeasy Plant Mini Kit (Qiagen) for RNA extraction. RNAs of both *Botrytis* species were sequenced at Beijing Genomic Institute (BGI), Shenzhen, China. Mapping and quantifying gene transcripts from sequenced RNA-seq reads of both *B. elliptica* and *B. squamosa* were performed using Kallisto (Bray et al., 2016). The +1 log10 TPM value was calculated for plotting the expression heatmap.

## Results

### Host specificity of *Botrytis* spp.

**Figure 1:**
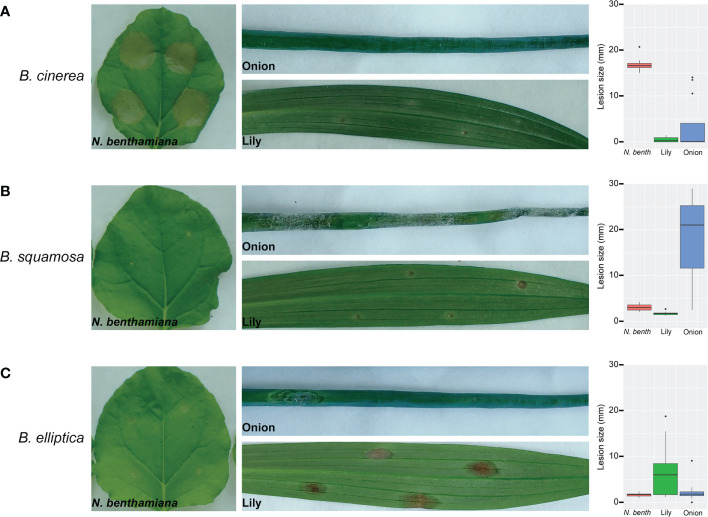
Inoculation of *B. cinerea* **(A)**, *B. squamosa* **(B)** and *B. elliptica* **(C)** on (*N. benthamiana*), onion and lily leaves confirms host-specificity. Disease symptoms were observed at 4dpi and box plot diagrams represent the lesion diameters at 4dpi upon inoculation of the different *Botrytis* species on the respective plants. Each box represents all compiled lesion diameters (in mm) for a given host plant.

*B. squamosa* and *B. elliptica* are reported to be host-specific pathogens of onion and lily, respectively. To assess this host specificity under laboratory conditions, we tested the virulence of *B. squamosa* and *B. elliptica* on their hosts and non-host plants and compared them to the broad host range pathogen *B. cinerea* (**Figure 1**). All three species were inoculated on onion, lily and *N. benthamiana* leaves and lesion development was monitored. The generalist *B. cinerea* was able to develop expanding lesions exclusively when inoculated on *N. benthamiana* leaves (**Figure 1A**) whereas the lesions on lily and onion leaves remained limited to the size of the inoculation droplet. Lesion expansion and disease progression were observed when *B. squamosa* was inoculated on onion leaves (**Figure 1B**) and when *B. elliptica* was inoculated on lily leaves (**Figure 1C**). In contrast, when *B. squamosa* and *B. elliptica* were cross-inoculated on lily and onion, respectively, the primary necrotic lesions did not expand over time and except for a few cases, they remained limited to the size of the inoculation droplet. In addition, inoculation of *B. squamosa* and *B. elliptica* on *N. benthamiana* leaves did not cause formation of expanding lesions. In conclusion, *B. squamosa* and *B. elliptica* caused expanding lesions on their reported natural host but not on the other tested plants.

### Novel genome sequence assembly and annotation

High molecular weight genomic DNA of *B. squamosa* and *B. elliptica* was isolated from mycelial cultures and was used for whole genome sequencing using Nanopore technology. Assembly of the reads resulted in genomes with a size of 55 and 50 Mb for *B. squamosa* and *B. elliptica*, respectively (**Table 1**). The assembly sizes are in accordance to the available genomes that were sequenced using PacBio technology as described in Valero-Jiménez et al. (2020) (55 and 48 Mb), but the quality of the assemblies was significantly improved. The new assemblies consisted of 19 and 26 contigs for *B. squamosa* and *B. elliptica*, respectively (**Table 1**), as compared to 29 and 137 contigs in the previously published genomes (Valero-Jiménez et al., 2020). Many contigs resembled complete chromosomes. For *B. squamosa* we assembled 10 out of the 19 contigs with telomeric repeats at both ends, while the remaining 9 contigs contained telomeric sequences at one end. For the genome of *B. elliptica*, we obtained 8 complete chromosomes containing telomeres at both ends.

**Table 1:** Assembly information of genomes of *B. elliptica* and *B. squamosa* sequenced in this study.

Species	#Contigs	Assembly size (Mb)	Largest contig (bp)	N50 (bp)	#Proteins	#Secreted proteins
*B. squamosa*	19	54.68	6112245	3203072	11933	897
*B. elliptica*	26	49.77	4879067	2914681	13585	925

Information regarding proteins and secretome was obtained using gene prediction tools.

**Figure 2:**
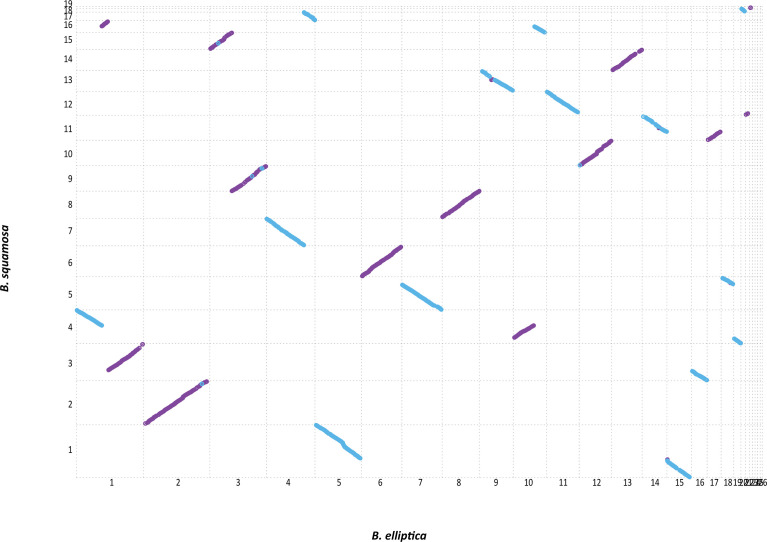
Synteny analysis of the genomes of *B. elliptica* and *B. squamosa* reveals a high level of synteny with few balanced structural rearrangements. Aligned contigs are color coded indicating the orientation of the synteny segments with purple indicating forward and blue indicating reverse orientation of the alignment.

A whole genome synteny analysis of *B. elliptica* and *B. squamosa* was performed by pairwise alignment of the assemblies. The genomes displayed a high level of synteny, with perfect alignment along 6 of the 16 contigs (**Figure 2**). There was evidence for 3 structural rearrangements between the contigs of *B. elliptica* and *B. squamosa.* For instance contig 1 of *B. elliptica* aligned to contig 3, 4, and 16 of *B. squamosa*, suggesting chromosomal rearrangements between the species. Similarly, contig 10 from *B. elliptica* aligned with contig 4 and 16 from *B. squamosa* (**Figure 2**).

The manually curated annotation of Valero-Jiménez et al. (2020) was used as input for annotation of the *B. squamosa* and *B. elliptica* genomes, together with RNAseq data from mycelial cultures and infected plant material. The genomes of *B. squamosa* and *B. elliptica* were predicted to contain 11,933 and 13,585 genes respectively, and we specifically examined and manually curated the gene models of the secretome (**Table 1**). The predicted secretome of *B. squamosa* consists of 897 genes (7.52%) while the predicted secretome of *B. elliptica* consists of 925 genes (6.81%) (**Table 1**). Based on the orthogroups described in Valero-Jiménez et al. (2020), we analyzed the predicted secretome for shared proteins between *B. squamosa* and *B. elliptica* and the more distantly related species *B. cinerea* which functions as the model species for the genus *Botrytis* (Pedro et al., 2019). The vast majority of the predicted secreted proteins could be assigned to 734 orthogroups that were shared between all three species (**Supplementary Figure 1**). A total of 91 orthogroups is shared only between *B. squamosa* and *B. elliptica* which reflects the recent divergence of these sister species as compared to *B. cinerea*. Only a small proportion of the predicted secretome is shared between *B. cinerea* and either *B. elliptica* or *B. squamosa* (31 and 20 orthogroups, respectively). Only 17, 26 and 55 predicted secreted proteins are unique for *B. squamosa*, *B. elliptica* and *B. cinerea*, respectively (**Supplementary Figure 1**; **Supplementary Data 1**).

The genome of *B. elliptica* isolate 9401 contains two mini-chromosomes (comprising both telomeres), of which the largest one is 199 Kb. These mini-chromosomes have no detectable synteny to the genome of *B. squamosa* (**Figure 2**). The largest mini-chromosome (contig 23, 199 kbp) contains 18 predicted genes, of which two appeared interesting. The gene BELL_9401_23g00040 encodes a predicted effector protein and has homologs in only *B. tulipae* (BTUL_0255g00010) and *B. hyacinthi* (BHYA_0337g00090) (Valero-Jiménez et al., 2019). The gene BELL_9401_23g00180 encodes a cysteine protease with potential ubiquitin-deconjugating activity, and it is orthologous to the gene Bcin18g00060 that is located on *B. cinerea* isolate B05.10 mini-chromosome Chr18. The second *B. elliptica* mini-chromosome (contig 25, 175 kbp) contains nine predicted genes, of which three have interesting features. BELL_9401_25g00050 has an ortholog in *B. cinerea* B05.10 on mini-chromosome Chr18 (Bcin18g00040, of unknown function), while BELL_9401_25g00060 encodes a putative Forkhead-domain protein kinase with a homolog in *B. cinerea* on mini-chromosome Chr17 (Bcin17g00060) and BELL_9401_25g00080 is orthologous to Bcin17g00070 (a putative ULP cysteine protease). All three genes appear to be expressed during *in vitro* growth and plant infection. The genome of *B. squamosa* includes a single mini-chromosome of 253 Kbp that contains 12 predicted genes. All of them have an ortholog in *B. elliptica* 9401 mini-chromosome (contig 22) but not in *B. elliptica* 9612 (Valero-Jiménez et al., 2020). Four of the 12 genes also have orthologs in *B. cinerea* B05.10, of which two are located on Chr9 and two on mini-chromosome Chr17. The orthologous *B. cinerea* genes on Chr9 encode a CAZyme GH43 protein and a secreted effector-like protein, whereas one of the genes on Chr17 (Bcin17g00060) encodes a Forkhead-domain protein kinase and is also orthologous to the gene in the *B. elliptica* mini-chromosome contig 25, as mentioned above.

### Liquid cultures of *Botrytis* spp. contain cell-death inducing proteins

**Figure 3:**
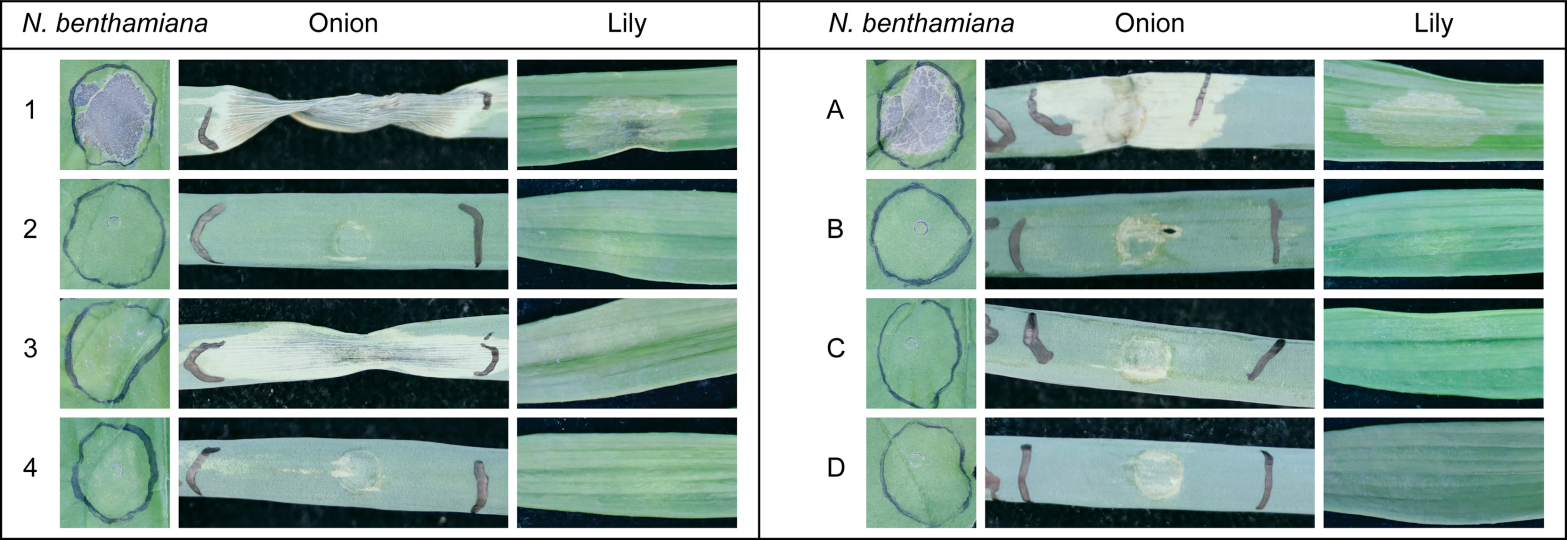
Leaf responses observed at 3dpi caused by infiltration of crude CF samples (left panel) and AS precipitated compounds (right panel) obtained from liquid cultures of *B. squamosa* and *B. elliptica* on leaves of *N. benthamiana*, onion and lily. 1 = *B. elliptica* grown in GB5_lily; 2 = *B. elliptica* grown in GB5_onion; 3 = *B. squamosa* grown in GB5_lily; 4 = *B. squamosa* grown in GB5_onion. **(A)** = AS precipitation of *B. elliptica* CF grown in GB5_lily; **(B)** = AS precipitation of *B. elliptica* CF grown in GB5_onion; **(C)** = AS precipitation of *B. squamosa* CF grown in GB5_lily; **(D)** = AS precipitation of *B. squamosa* CF grown in GB5_onion.

In order to analyze the cell death-inducing activity of proteins secreted by the different *Botrytis* spp., we collected culture filtrate (CF) samples from *B. squamosa* and *B. elliptica* grown in liquid cultures. The CFs were infiltrated into *N. benthamiana*, onion and lily to assess plant cell death responses. Plant responses were recorded at 3 dpi (**Figure 3**) and red light imaging (Villanueva et al., 2021) was used to visualize plant cell death responses (**Supplementary Figure 2**). *B. elliptica* grown in presence of lily leaf extract, caused the most severe response in all tested plant species. Upon infiltration of this sample (CF number 1 in **Figure 3**, left panel), we observed collapse of the tissue, drying and intense yellowing/browning at the infiltrated area. By contrast, leaf infiltration with compounds collected from *B. elliptica* grown in presence of onion leaf extract (CF number 2 in **Figure 3** left panel) caused a milder response and only in lily leaves, where the infiltrated tissue showed a slight translucence. On the other hand, compounds collected from *B. squamosa* grown in presence of lily leaf extract (CF number 3 in **Figure 3**, left panel) caused a more severe response when compared to the compounds collected from *B. squamosa* grown in presence of onion leaf extract (CF number 4 in **Figure 3**, left panel). Both *B. squamosa* CF samples did not cause any clear response upon infiltration in lily and *N. benthamiana* leaves, although in the highly sensitive red light imaging system some cell death response could still be observed (**Supplementary Figure 2**). When CF samples from *B. squamosa* grown in the presence of lily leaf extract were infiltrated in onion leaves, tissue collapse, drying and intense yellowing was observed. Infiltration of the medium used for the fungal liquid culture caused no detectable response in any of the tested plants (**Supplementary Figure 2**). CF samples contain proteins and secondary metabolites secreted by the fungus, both of which potentially possess cell death-inducing activities. To distinguish the proteinaceous activity from the activity of other compounds, ammonium sulfate precipitation was performed to concentrate the proteins secreted by the fungi in the liquid medium. After dialysis of the protein pellet, the resuspended precipitates were infiltrated in *N. benthamiana*, onion and lily leaves. The ammonium sulfate precipitation of the proteins did not notably affect the cell death inducing activity of *B. elliptica* proteins, since the precipitated proteins derived from *B. elliptica* liquid culture grown in presence of lily leaf extract showed the strongest cell death inducing activity in all tested plants (sample A in **Figure 3**, right panel; **Supplementary Figure 3**). The symptoms appeared similar to those observed upon infiltration of the crude CF. In contrast, ammonium sulfate precipitation of the proteins contained in *B. squamosa* CF sample grown in presence of lily leaf extract substantially lost their cell death inducing activity when infiltrated in onion leaves (**Figure 3**, right panel sample C; **Supplementary Figure 3**). This result suggests that the cell death-inducing activity of the culture filtrate does not originate from proteins but from phytotoxic secondary metabolites such as botrydial, for which a functional biosynthetic gene cluster is present in *B. squamosa* (Colmenares et al., 2002; Valero-Jiménez et al., 2020). Infiltration of the dialysis buffer caused no visible response in any of the plants tested (**Supplementary Figure 3**).

### Protein composition of the cell death-inducing culture filtrates

**Figure 4:**
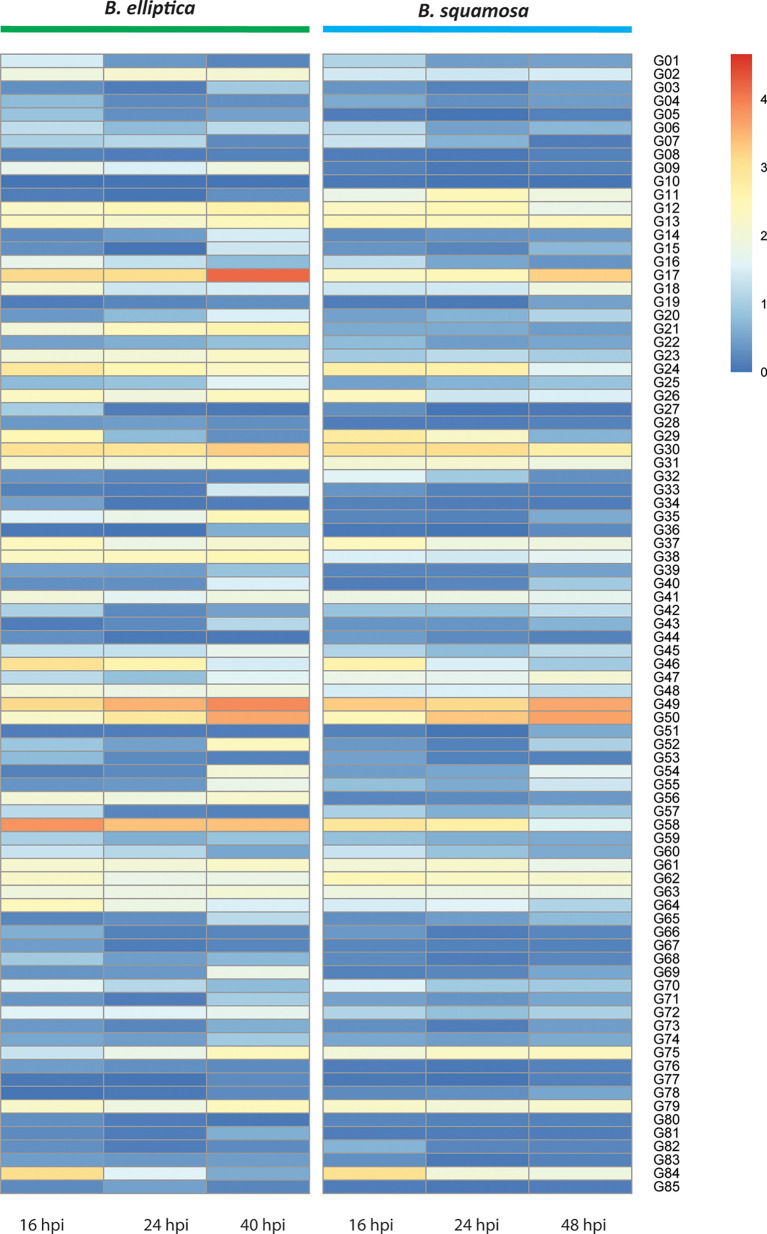
Heatmap showing expression profiles of genes of which the proteins were identified in the CF samples of *B. elliptica* and *B. squamosa*. Genes from both species that belong to the same orthogroups are aligned horizontally. Expression is based on the average of three replicate RNAseq samples during different timepoints of infection of *B. elliptica* and *B. squamosa* on their respective host plants.

The identity of proteins present in the CF samples that showed cell death-inducing activity was analyzed using mass spectrometry. The *B. elliptica* CF sample contained significant hits for a total of 165 proteins identified in the predicted secretome. More specifically, we detected 70 CAZymes (42% of total number), 15 putative effectors, 8 oxidoreductases and 24 proteases (14,5%). The remaining proteins represented enzymes related to lipid metabolism, different phosphatases, sugar and ion transporters, or proteins without a described domain. In the *B. squamosa* CF sample, 114 proteins with a predicted signal peptide could be identified. Compared to the CF of *B. elliptica*, the *B. squamosa* CF sample contains a considerably higher proportion of CAZymes (69 correspondig to 60% of the total number) but a lower proportion of proteases (7%) and only three putative effectors. In total 85 proteins are present both in *B. elliptica* and *B. squamosa* CF samples (**Figure 4**). Only three proteins present in both CF samples are predicted to be putative effectors: G17 (orthologs to *B. cinerea* Spl1, Frías et al., 2011) and G58, a small (16KDa) cysteine rich protein of unknown function. Both CFs contained a protein with a chorismate mutase domain that also carries a predicted chloroplast targeting peptide (G63). Interestingly, the orthologous region in *B. cinerea* appeared to be pseudogenized since it carries a point mutation in the start codon.

To investigate the potential contribution to virulence of the 85 shared proteins detected in the CF samples, we analyzed the transcript levels of their corresponding genes at different timepoints during host infection (**Supplementary Data 2**). **Figure 4** shows a heatmap of the gene expression profile of orthologous genes of *B. squamosa* and *B. elliptica* during infection. When examining the transcript levels in the two *Botrytis* spp., there are three orthogroups with relatively high expression at 40-48 hpi (G17, 49 and 50). For example,G17 which is orthologous to the *B. cinerea* cell death-inducing protein Spl1 (Frias et al., 2011) and G49 which encodes an aspartic protease, both peaked in expression in both species at 40-48 hpi. In contrast, some orthogroups had the highest relative expression at 16 hpi (G24, G29, G46, 58, 62 and 84) in both *B. squamosa* and *B. elliptica*. G24 encodes a putative endoglucanase (GH12) and G58 is predicted as a putative effector. Some orthologs showed different expression patterns between the two fungi, such as G09, G11, G18, G21, G23, G35, G38, G58. For instance, the orthogroup G21, which encodes a polygalacturonase also found in *B. cinerea*, was relatively highly expressed throughout infection in *B. elliptica*, but not in *B. squamosa.* spp.

## Discussion

Previously the genomes of 16 *Botrytis* spp. with distinct host specificity were analyzed in order to identify unique genes that might encode virulence factors associated to host specificity (Valero-Jiménez et al., 2020). However, no specific elements were found, suggesting that host specificity may be determined by a combination of different (quantitative) traits rather than by presence or absence of unique virulence factors. The aim of this study was to analyse shared features of the secretome in *B. elliptica* and *B. squamosa* that contribute to cell death induction and virulence on their host and non-host plants. This was done by comparing their genomes and by testing the cell death-inducing activity of their secreted proteins.

Genome sequences were available for both species (Valero-Jiménez et al., 2020). However, we considered it useful to further improve the assembly of the *B. squamosa* reference genome and to newly sequence a different *B. elliptica* isolate 9401 (Malvestiti et al., 2021) that was much more virulent than the isolate *B. elliptica* 9612 previously sequenced by Valero-Jiménez et al. (2020). The new genome assemblies of *B. elliptica* 9401 and *B. squamosa* MUCL31421 indeed provided significant improvement over the previous assemblies and enabled us to perform a whole genome alignment and synteny analysis between *B. squamosa* and *B. elliptica.* They display a high degree of synteny, with evidence for only three balanced chromosomal rearrangements. A reconstruction of the ancestral genome of the genus *Botrytis* by Valero-Jiménez et al. (2020) had provided indications for the occurrence of very low numbers of balanced chromosomal rearrangements between the common ancestor of the genus *Botrytis* and the extant species, despite the estimate that they diverged ~5 Million years ago (Shen et al., 2020). The chromosome configurations of *B. squamosa* and *B. elliptica* provide further indications for the remarkable stability of genomes in the genus *Botrytis*, as proposed by Valero-Jiménez et al. (2020). Moreover, the manually curated annotations by Valero-Jiménez et al. (2020) could largely be transferred to the new assemblies of *B. squamosa* and *B. elliptica*, and provided a reliable insight into their proteome and especially secreted protein repertoires. The predicted secretomes of *B. squamosa* and *B. elliptica* were more similar to each other than either of the two species resembled the *B. cinerea* secretome, in concordance with their recent divergence and larger evolutionary distance to *B. cinerea*.

Interestingly, the genome assemblies yielded evidence for the occurrence in both species of mini-chromosomes with lengths of 175-253 kb. One mini-chromosome was identified in *B. squamosa* and two in *B. elliptica.* These mini-chromosomes contain few (predicted) genes, however, remarkably they contain orthologs of genes that are also present on the mini-chromosomes of *B. cinerea*, which is believed to have diverged from *Botrytis* species in clade 2 around 5 Million years ago (Shen et al., 2020). Intriguingly, homologs of several genes on *B. elliptica* and *B. squamosa* mini-chromosomes were detected in distant species (*B. tulipae and B. hyacinthi*) but none were detected in the close relatives *B. deweyae* and *B. sinoalli*, suggesting that certain more distant *Botrytis* species may possess homologous mini-chromosomes, while related species might not possess them. One gene that seems to be shared among mini-chromosomes in *B. cinerea*, *B. elliptica* and *B. squamosa* encodes a putative Forkhead-domain protein kinase, while other genes that are shared between two of the three species include genes encoding a putative effector, a glycosyl hydrolase (GH43) and a cysteine peptidase with potential ubiquitin-deconjugating activity. The presence of such genes on mini-chromosomes of three distinct species might explain their retention in the species and the population. The function of these genes in the biology and virulence of these species clearly deserves further analyses.

Besides genome comparisons, we tested the cell death-inducing activity of *B. elliptica* and *B. squamosa* proteins secreted during liquid culture. The CF sample of *B. elliptica* contained a much larger diversity of proteins than *B. squamosa*. Upon leaf infiltration, the *B. elliptica* CF sample caused cell death not only in lily, but also in onion and *N. benthamiana* (**Figure 3**). On the other hand, the *B. squamosa* CF samples only caused severe necrotic responses in onion leaves. These observations suggest that the larger diversity and the combined activity of *B. elliptica* secreted proteins might confer the capability of causing cell death not only in lily (Liliaceae), its natural host, but also in phylogenetically distant plant species such as *N. benthamiana* (Solanaceae) and onion (Amaryllidaceae). This hypothesis is supported by the fact that cell death induction and plant susceptibility in plant-necrotroph interactions often correlate in a quantitative manner. As shown for the wheat-*P. nodorum* interaction, the quantitative level of plant susceptibility is correlated to the number of cell death-inducing effectors that are produced by the fungus and the number of effector target loci present in the host (Tan et al., 2012; Haugrud et al., 2019). One should also consider that during fungal infection, cell death-inducing proteins are gradually expressed and secreted by the fungus and their levels must be sufficiently high to induce cell death responses that indeed facilitate fungal colonization. Experiments with CF samples can only partly mimic the events occurring during plant-necrotroph interactions.

CF samples that could induce host cell death on onion or lily contained subsets of (predicted) effectors as well as CAZymes. The samples of *B. elliptica* and *B. squamosa* contained proteins that could be grouped into 85 orthogroups shared between the species. Not all genes in these orthogroups were expressed during host infection, suggesting that they may have different roles in the biology of these pathogens. Based on transcriptome data of *B. elliptica*-infected lily and *B. squamosa*-infected onion, there were 15-20 genes in each of the species that appeared to display significant expression, including a subset of genes that displayed a transient expression peak in early phases of infection. Especially proteins from G58, encoding a putative effector, and G17 which encodes orthologs of the *B. cinerea* cell death-inducing protein Spl1 (Frías et al., 2011) are interesting candidates for analysis.

Both CF samples from *B. squamosa* and *B. elliptica* contained proteins that were uniquely present in that sample. Since the focus of this study is on the shared cell death inducing proteins between the two *Botrytis* spp., these unique proteins were not analysed. However, such unique proteins showing high gene expression during infection can be further investigated to explore their contribution to virulence in the respective host plants. This list still comprises a few dozen genes, and experiments are ongoing to purify CF samples through ion exchange chromatography and to test the activity of fractions for cell death-inducing activity. These efforts should result in reducing the number of candidate genes to a manageable number for functional analysis through CRISPR/Cas9-mediated deletion (Leisen et al., 2020; Leisen et al., 2022).

## Data availability statement

The Whole Genome Shotgun project of *B. elliptica* 9401 has been deposited at DDBJ/ENA/GenBank under the accession JANCTG000000000. The version described in this paper is version JANCTG010000000. The Whole Genome Shotgun project of *Botrytis squamosa* MUCL31421 has been deposited at DDBJ/ENA/GenBank under the accession RCTC00000000. The version described in this paper is version RCTC02000000.

## Author contributions

MM and MS contributed equally to this work. MM, MS, XS-K and JK designed the study. MM, MS, HB and SB performed the lab experiments and analysed the data. XS-K performed the genome assemblies and computational analyses. MM, MS, XS-K and JK wrote the manuscript. All authors contributed to the article and approved the submitted version.

## Funding

The research of MCM is funded by NWO-Science domain (NWO-ENW), project GSGT.2018.008. The research of MBFS was financially supported by the Dutch Technology Foundation TTW, which is part of the Netherlands Organisation for Scientific Research (NWO) and which is partly funded by the Ministry of Economic Affairs (project 15003).

## Acknowledgments

The plant material used was kindly provided by De Jong Lelies, Hobaho, Mak Breeding, Royal van Zanten, Vletter-den Haan, and World Breeding. The authors are grateful to Rinie Verwoert, Rohan van Genderen, and Wilfred Krijnen for their support in cultivation of the lilies.

## Conflict of interest

The authors declare that the research was conducted in the absence of any commercial or financial relationships that could be construed as a potential conflict of interest.

## Publisher’s note

All claims expressed in this article are solely those of the authors and do not necessarily represent those of their affiliated organizations, or those of the publisher, the editors and the reviewers. Any product that may be evaluated in this article, or claim that may be made by its manufacturer, is not guaranteed or endorsed by the publisher.

## Supplementary material

Supplementary Information 1 Detailed procedure for protein preparation for Mass Spectrometry analysis.

Supplementary Data 1 Detailed information of orthogroups, and Interproscan results of specific groups of proteins.

Supplementary Figure 1 Secretome conservation of three *Botrytis* spp. Presence or absence of secreted protein genes among *B. cinerea*, *B. elliptica* and *B. squamosa* are indicated by dark and light blue, respectively.

Supplementary Figure 2 Leaf responses observed at 3dpi caused by infiltration of crude CF samples obtained from liquid cultures of *B. squamosa* and *B. elliptica* on leaves of *N. benthamiana*, lily and onion visualized with normal light and with the red light imaging system. 1 = *B. elliptica* grown in GB5_lily; 2 = *B. elliptica* grown in GB5_onion; 3 = *B. squamosa* grown in GB5_lily; 4 = *B. squamosa* grown in GB5_onion; 5 = GB5_onion mock; 6 = GB5_lily mock.

Supplementary Figure 3 Leaf responses observed at 3dpi caused by infiltration of AS precipitated compounds contained in the CF samples in *N. benthamiana*, lily and onion visualized with normal light and with the red light imaging system. A = AS precipitation of *B. elliptica* CF grown in GB5_lily; B = AS precipitation of *B. elliptica* CF grown in GB5_onion; C = AS precipitation of *B. squamosa* CF grown in GB5_lily; D = AS precipitation of *B. squamosa* CF grown in GB5_onion; E = mock dialysis buffer.

Supplementary Data 2 Expression levels and functional annotation of the 85 shared proteins detected in the culture filtrates of *B. squamosa* and *B. elliptica*.
