# Supplementary material for: Analysis of plant cell death-inducing proteins of the necrotrophic fungal pathogens Botrytis squamosa and Botrytis elliptica
Source: Front Plant Sci. 2022 Oct 11;13:993325. doi: 10.3389/fpls.2022.993325 (PMC9593002; doi:10.3389/fpls.2022.993325)
Supplement: Supplementary Information 1 — Detailed procedure for protein preparation for Mass Spectrometry analysis. [file DataSheet_3.docx]

**Supplementary Information:**

**Detailed procedure for protein preparation for Mass Spectrometry analysis**

Proteins present in 1mL crude CF sample were precipitated with 20% trichloroacetic acid and washed with 80% acetone. The precipitated pellets were boiled for 10 minutes in 20 μL Laemmli buffer (4% SDS; 20% glycerol; 125 mM Tris-HCl, pH 6.8), loaded on a precast stain free protein gel (4–20% Mini-PROTEAN TGX Stain-Free, Biorad) and run for 15 min at 70V. After running the gel was stained with Coomassie Brilliant Blue (H_2_O 50%; 40% EtOH; 10% AcOH; 0,1% CBB R250). The gel was kept for ca 30 min on the shaker for staining, washed two times with de-staining solution (H_2_O/ EtOH/ AcOH 60:30:10 v/v/v) and stored in 50% MeOH at 4°C. Stained protein bands were cut with a scalpel in ca 1 mm2 pieces and transferred to a clean 0.5mL protein low binding tube. Then reduction of cysteine disulfide bridges took place by adding 100μL 10 mM dithiothreitol (DTT, 1.5mg/mL) in 50 mM ammonium bicarbonate (ABC) pH=8 and one hour shaking at 45°C. After cooling down to room temperature DDT was replaced by 100μL 15 mM acrylamide (1.1mg/mL) in 50 mM Tris (pH=8) for protein alkylation and the samples were shake incubated 30 minutes at 20°C. Subsequently the acrylamide was washed out two times with 50 mM ABC pH=8 and the samples were frozen to facilitate trypsin digestion. Trypsin digestion took place by adding to the samples 100μL fresh prepared trypsin solution (5 ng/μL trypsin in 50mM ABC pH=8) and after overnight incubation on the shaking plate at 20°C. Two pieces of C18 Empore disk (3M Empore C18 Extraction Disks, Fisher) were mildly tapped into a clean 200μL pipet tip and washed with 200μL MeOH. A 50% slurry of LichoprepC18 column matrix (LiChrosorb RP-8 HPLC Column, Merk) in MeOH was prepared and 4μL of the 50% slurry were added into the MeOH containing 200μL pipet tip. Without letting the microcolumn material dry out, the 200μL MeOH were pressed out with a 10mL plastic syringe and fresh 100μL were added. The column material was equilibrated once with 100μL 1mL/L formic acid in water and the tryptic peptides were dissolved in 100μL formic acid. The sample was added to the microcolumn and the gel pieces were washed once extra with 100μL formic acid and add to the microcolumn. The formic acid was then eluted with a syringe, 100μL fresh formic acid were added to the microcolumn and eluted. The microcolumn was transferred to a protein low binding 0.5mL tube (Thermofisher) and 50μL of 50% acetonitrile in formic acid were added to the microcolumn. Tryptic peptides bound to the column matrix were eluted with a syringe into a 0.5mL protein low binding tube. The samples were then dried in a vacuum concentrator (Eppendorf) at 45 °C for 30 min until the volume of each sample decreased to 15μL, and stored at –20 °C.
