# Supplementary figures and images for: Analysis of plant cell death-inducing proteins of the necrotrophic fungal pathogens Botrytis squamosa and Botrytis elliptica
Source: Front Plant Sci. 2022 Oct 11;13:993325. doi: 10.3389/fpls.2022.993325 (PMC9593002; doi:10.3389/fpls.2022.993325)

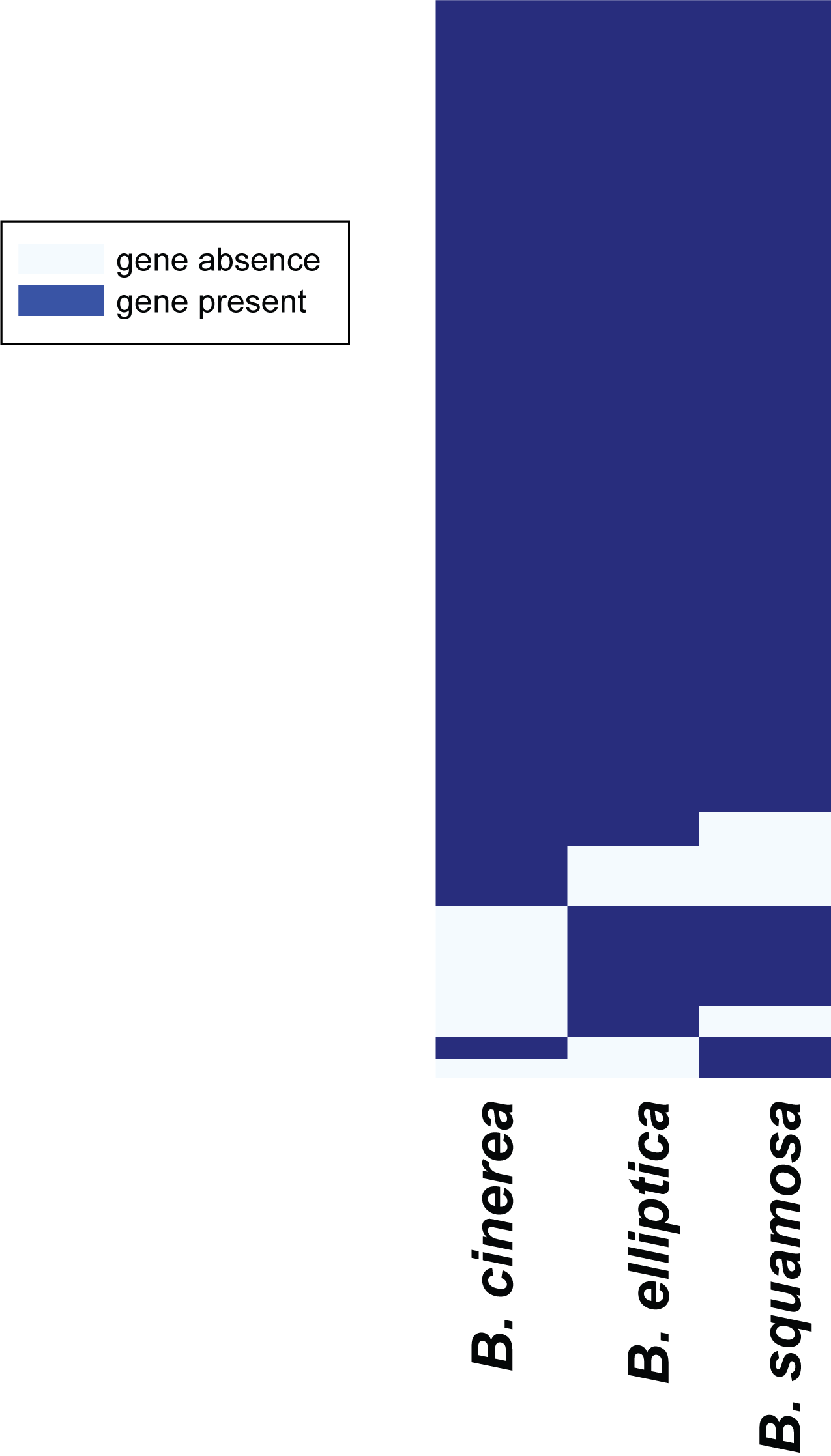

Supplement: Supplementary Figure 1 — Secretome conservation of three Botrytis spp. Presence or absence of secreted protein genes among B. cinerea, B. elliptica and B. squamosa are indicated by dark and light blue, respectively. [file Image_1.tif]

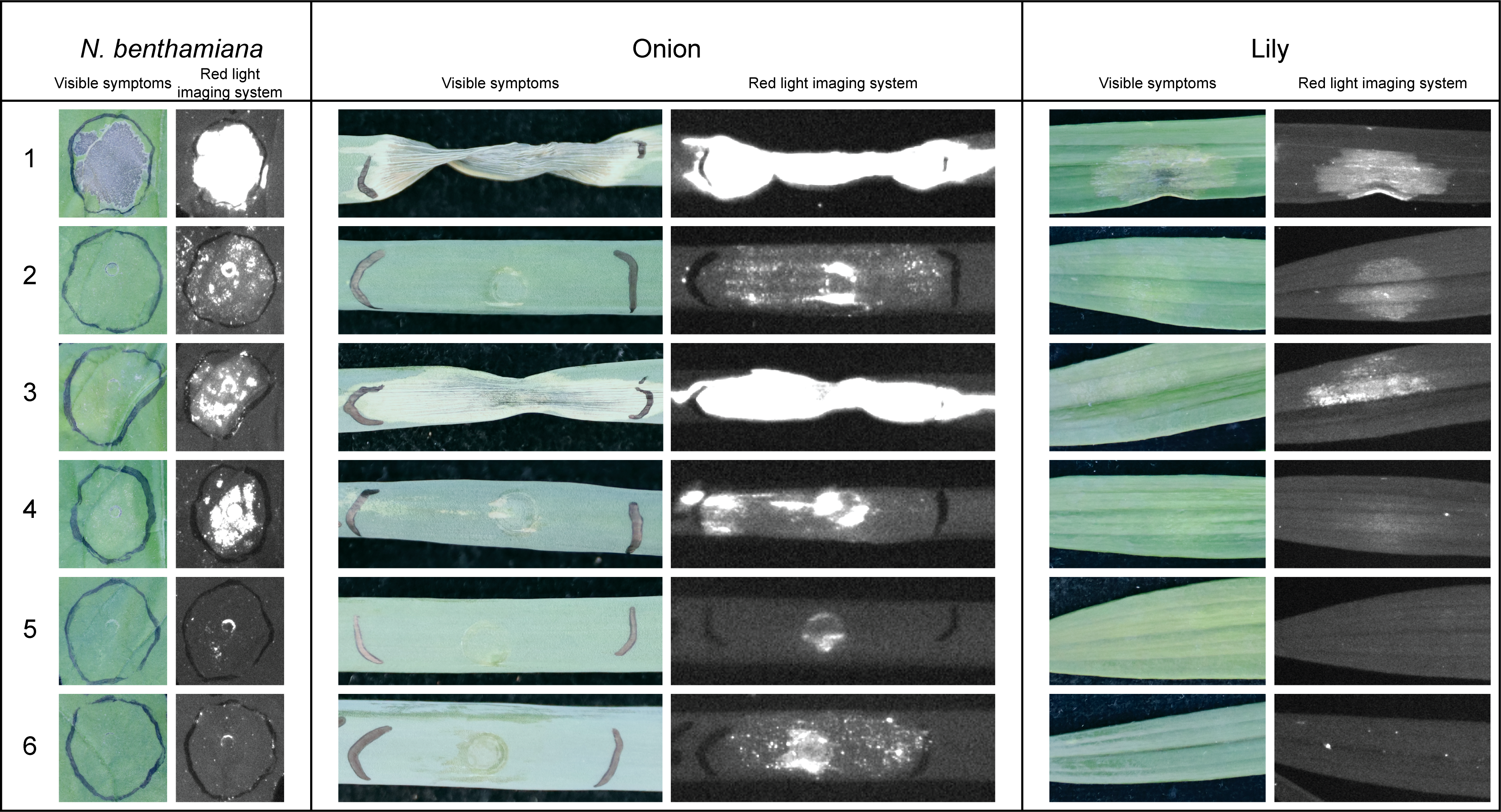

Supplement: Supplementary Figure 2 — Leaf responses observed at 3dpi caused by infiltration of crude CF samples obtained from liquid cultures of B. squamosa and B. elliptica on leaves of N. benthamiana, lily and onion visualized with normal light and with the red light imaging system. 1 = B. elliptica grown in GB5_lily; 2 = B. elliptica grown in GB5_onion; 3 = B. squamosa grown in GB5_lily; 4 = B. squamosa grown in GB5_onion; 5 = GB5_onion mock; 6 = GB5_lily mock. [file Image_2.tif]

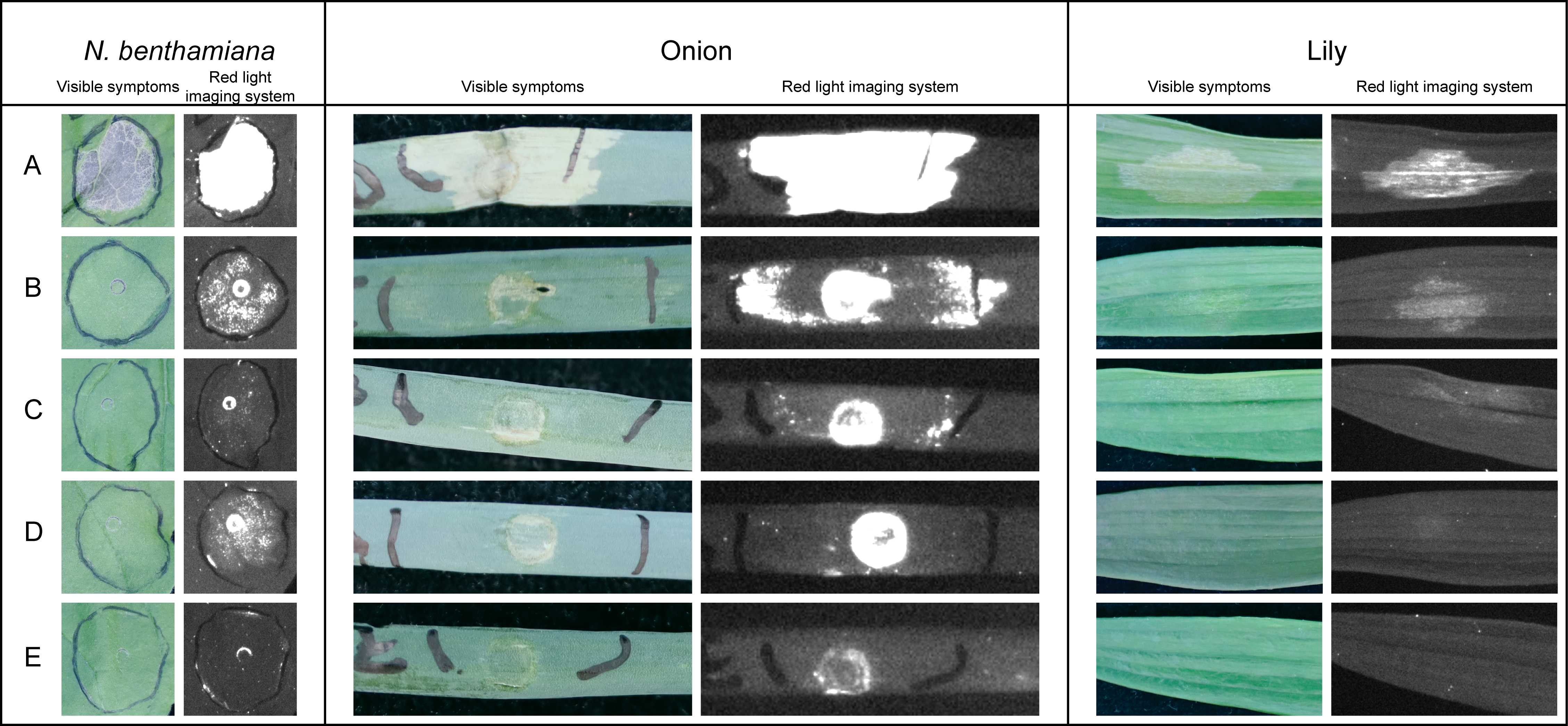

Supplement: Supplementary Figure 3 — Leaf responses observed at 3dpi caused by infiltration of AS precipitated compounds contained in the CF samples in N. benthamiana, lily and onion visualized with normal light and with the red light imaging system. A = AS precipitation of B. elliptica CF grown in GB5_lily; B = AS precipitation of B. elliptica CF grown in GB5_onion; C = AS precipitation of B. squamosa CF grown in GB5_lily; D = AS precipitation of B. squamosa CF grown in GB5_onion; E = mock dialysis buffer. [file Image_3.tif]
